# Comparative transcriptomic analyses of glucosinolate metabolic genes during the formation of Chinese kale seeds

**DOI:** 10.1186/s12870-021-03168-2

**Published:** 2021-08-21

**Authors:** Yijiao Zhao, Zeyuan Chen, Jiaxuan Chen, Bingxing Chen, Weiling Tang, Xiaodong Chen, Zhongxiong Lai, Rongfang Guo

**Affiliations:** 1 College of Horticulture, Institute of Horticultural Biotechnology, Fujian Agriculture and Forestry University, Fuzhou, 350002 China; 2 Joint FAFU-Dalhousie Lab, College of Horticulture, Fujian Agriculture and Forestry University, Fuzhou, 350002 China

**Keywords:** Glucosinolates, Seed, Chinese kale, *AOP2*, *beta-glucosidases*, *GTR*

## Abstract

**Background:**

To understand the mechanism of glucosinolates (GSs) accumulation in the specific organs, combined analysis of physiological change and transcriptome sequencing were applied in the current study. Taking Chinese kale as material, seeds and silique walls were divided into different stages based on the development of the embryo in seeds and then subjected to GS analysis and transcriptome sequencing.

**Results:**

The main GS in seeds of Chinese kale were glucoiberin and gluconapin and their content changed with the development of the seed. During the transition of the embryo from torpedo- to the early cotyledonary-embryo stage, the accumulation of GS in the seed was accompanied by the salient decline of GS in the corresponding silique wall. Thus, the seed and corresponding silique wall at these two stages were subjected to transcriptomic sequencing analysis. 135 genes related to GS metabolism were identified, of which 24 genes were transcription factors, 81 genes were related to biosynthetic pathway, 25 genes encoded catabolic enzymes, and 5 genes matched with transporters. The expression of GS biosynthetic genes was detected both in seeds and silique walls. The high expression of *FMOGS-OX* and *AOP2*, which is related to the production of gluconapin by side modification, was noted in seeds at both stages. Interestingly, the expression of GS biosynthetic genes was higher in the silique wall compared with that in the seed albeit lower content of GS existed in the silique wall than in the seed. Combined with the higher expression of transporter genes *GTRs* in silique walls than in seeds, it was proposed that the transportation of GS from the silique wall to the seed is an important source for seed GS accumulation. In addition, genes related to GS degradation expressed abundantly in the seed at the early cotyledonary-embryo stage indicating its potential role in balancing seed GS content.

**Conclusions:**

Two stages including the torpedo-embryo and the early cotyledonary-embryo stage were identified as crucial in GS accumulation during seed development. Moreover, we confirmed the transportation of GS from the silique wall to the seed and proposed possible sidechain modification of GS biosynthesis may exist during seed formation.

**Supplementary Information:**

The online version contains supplementary material available at 10.1186/s12870-021-03168-2.

## Background

Chinese kale (*B. oleracea*), a member of the Cruciferae, is notable for its high content of glucosinolates (GS), which show excellent health-promoting properties, as well as rich contents of carotene and vitamin C [1]. Chinese kale is a vegetable crop that originated in southern China and is a well-known specialty vegetable in China, with a crisp, tender, and unique flavor [2–4]. Glucosinolates are a class of steroid glycosides synthesized from glucose and amino acids. These compounds widely occur as secondary metabolites in cruciferous species, especially *Arabidopsis* and a large number of economically valuable vegetables [5–7]. Based on the amino acids from which the compounds are derived, GS can be categorized into aliphatic GS, indolic GS, and aromatic GS [8, 9].

In plants, GS localized within the vacuoles of specific cells [10]. Upon herbivore damage, GS mix with the enzyme myrosinase (EC3.2.1) leading to the formation of breakdown products [11]. The hydrolysis of indolic GS leads to the formation of unstable isothiocyanates (ITCs) and nitriles, whereas aliphatic and aromatic GS mostly produce noxious ITCs [12]. Different GS groups endow plants with different resistance against distinct attackers. For example, indolic GSs act against phloem feeders and pathogens [13], whereas aliphatic, indolic, and benzyl GS may affect the performance of chewing insects [14].

The GS content and profiles of GS are exceedingly diverse in different Chinese kale varieties [15]. GSs are constitutively present in all tissues of brassicaceous plants, but differentially distributed over different organs, among which reproductive organs (e.g., seeds, pods, and developing inflorescences) have the highest GS content, followed by young leaves and roots [16]. The accumulation of GS is a complex process that may be regulated by multiple mechanisms [17, 18]. The mediators that regulate GS accumulation are mainly related to three aspects of the process, that is: i) GS biosynthesis, ii) GS degradation, and iii) GS transportation. In the model plant *Arabidopsis*, the majority of GS biosynthetic and degradation genes have been identified [19–21]. The synthesis of GS in *Arabidopsis* involves three independent processes: elongation of specific amino acids, formation of the core structure, and secondary modification of side chains [17, 22]. After comparison with the *Arabidopsis* genes, GS biosynthetic homologous genes were identified in Chinese kale sprouts in our previous study [3]. The degradation of GS in plants may also exert an important influence on GS accumulation and is mediated by catabolic enzymes [6]. Myrosinase, also known as β-thioglucosidase, is a hydrolytic enzyme commonly present in cruciferous plants that efficiently degrade GSs [11, 23, 24]. Six myrosinase genes (THIOGLUCOSIDE GLUCOHYDROLASE 1–6; *TGG1*-*TGG6*) have been identified in *Arabidopsis* [25–27]. In addition, *PENETRATION 2* (*PEN2*) and *PYK10* are capable of hydrolyzing indolic GS in *Arabidopsis* [28, 29]. Recently, more than half of the β-thioglucosidases in *Arabidopsis* were shown to exhibit myrosinase activity (BGLU18-BGLU39) [29, 30]. In addition, transport processes are important for the reallocation of defensive compounds to protect tissues of high value in plants. As demonstrated in *Arabidopsis*, GSs are translocated to seeds at maturation by *NITRATE TRANSPORTER 1/PEPTIDE TRANSPORTER* (*NRT1/PTR*) family transporters [31–33]. The *NRT1/PTR* family includes *NPF2.10/GTR1*, *NPF2.11/GTR2*, and *NPF2.9/GTR3*. Among these transporters, GTR1 and GTR2 are widely considered to show GS transport activity, whereas GTR3 specifically transports the indolic GS 3-ylmethyl glucosinolate [32, 34, 35].

Vegetable crops harbor a greater number of homologous genes associated with GS biosynthesis than those identified in *Arabidopsis* [36]. However, in Chinese kale, the homologs that play a crucial role in a specific metabolic process remain unknown, rendering it impossible to utilize gene editing for the improvement of vegetable quality. Transcriptome sequencing (RNA sequencing) is an efficient and widely used technique for acquiring deep transcriptome information and achieving a thorough understanding of biological transcripts, especially those involved in a metabolic pathway in a specific tissue. Simultaneously, RNA sequencing analysis is used to quantify transcript levels and also enables the identification of novel transcripts to improve the annotation of a genome [37]. In a previous study, we have identified genes related to GS metabolism in Chinese kale sprouts [3]. However, with the aim to improve the GS content in Chinese kale sprouts, we observed that the GS content in sprouts is the result of seed release, biosynthesis, degradation, and transport, among which seed release is a dominant factor affecting GS accumulation in sprouts [3]. Therefore, an increase in the seed GS is crucial to regulate the GS content of Chinese kale sprouts.

The distribution of GS over different parts follows optimal defense theory which allows plants to allocate defense compounds preferentially to the valuable plant parts which are also attractive to potential attackers [38]. After domestication, varieties with high-value edible parts are selected, complicating the composition of GS in seeds. The in silico analysis of GS biosynthetic gene expression in Arabidopsis indicated the important role of GS allocation from silique walls to seeds [17, 24]. However, in Chinese kale, which contains different GS profiles compared with Arabidopsis, knowledge of sources for GS accumulation accompanying seed development is still limited. In the present study, we observed the decline of GS content in the silique wall and the increasing accumulation of GS in the seed during the development of the embryo in Chinse kale. Thus, we proposed that the accumulation of GS in the seed may be related to the transportation of GS from silique walls. By physiological analysis of GS accumulation with development of the seed and corresponding silique wall, we have identified the crucial stages for GS transition, that was torpedo- and cotyledonary-embryo, and these two stages were subjected to the transcriptome analysis after RNA sequencing. Finally, we concluded possible sidechain modification of GS may occur during seed formation in Chinese kale.

## Results

### Embryo development during Chinese kale seed formation and accumulation of GS in the seed and corresponding silique wall

**Fig. 1:**
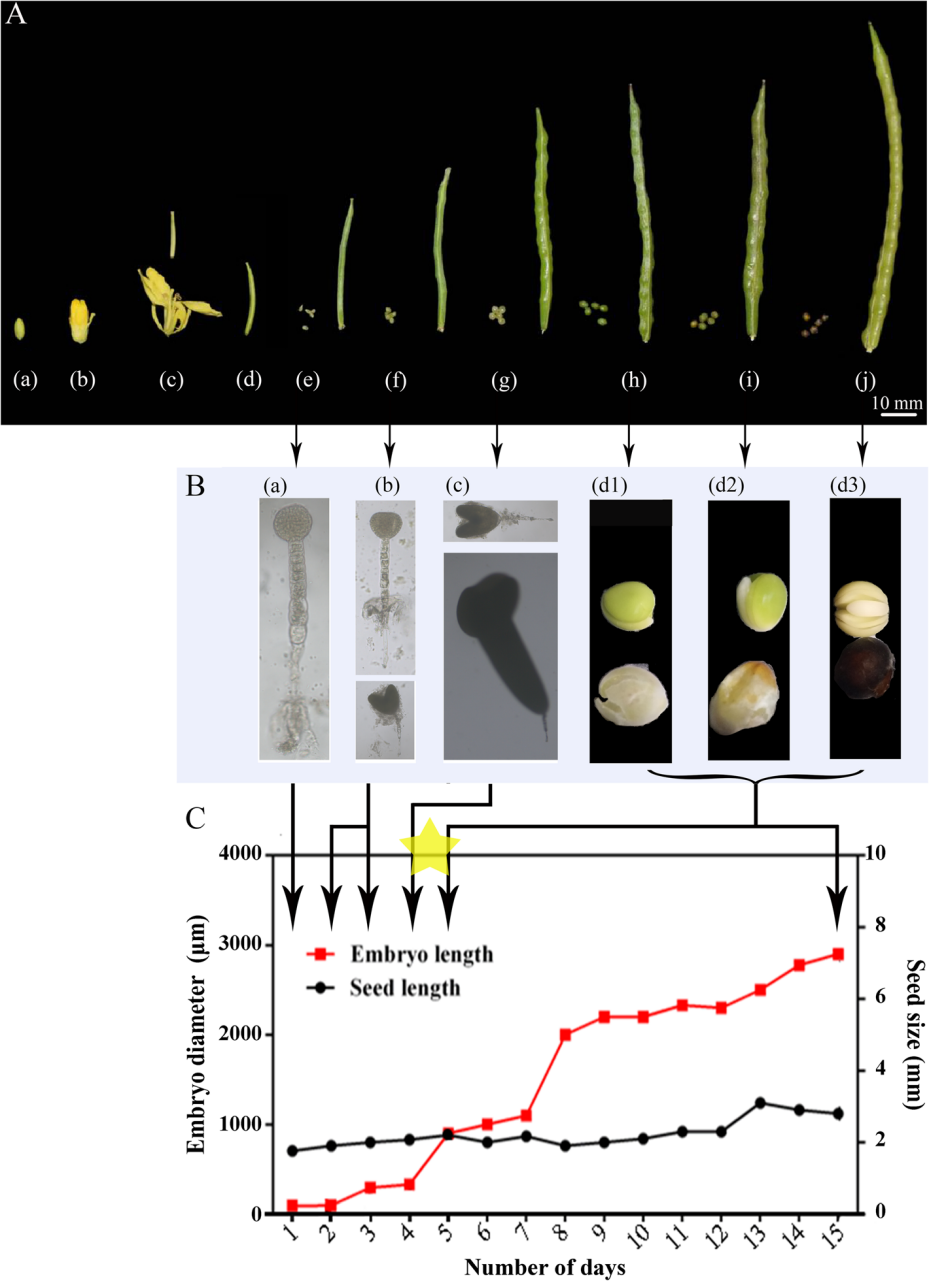
Development of the seed and corresponding silique as well as the morphogenesis of embryo during seed formation in Chinese kale. **A** Development of the seed and corresponding silique in Chinese kale*.* (a) Small flower bud (7 DBF), (b) Big flower bud (1 DBF), (c) 10 mm silique with seeds (up) and flower (down) (day 0), (d) 20 mm silique with seeds (3 DAF), (e) 35 mm silique with seeds (9 DAF), (f) 40–50 mm silique with seeds (15 DAF), (g) 55–65 mm silique with seeds (31 DAF), (h) Green silique with seeds (41 DAF), (i) Semi-brown silique with seeds (48 DAF), (j) Full brown silique with seeds (53 DAF). DAF stands for day before flower and DAF stands for day after flower. **B** Morphogenesis of embryo during the formation of seed in Fig. 1A. (a) Globular embryo, (b) Heart-shaped embryo, (c) Torpedo-shaped embryo, (d1) Early cotyledonary embryo and seed coat, (d2) Medium cotyledonary embryo and seed coat, (d3) Late cotyledonary embryo and seed coat. **C** Change of embryo diameter and seed size during seed formation. Three biological replicates were used for the measurements

After the emergence of the flower bud, the entire process of floral and fruit development was tracked (Fig. 1A). Ten days after bud emergence, the flower was fully open and the silique was 10 mm in length (day 0). The silique grew quickly to a length of 35 mm at 9 days after flowering (DAF) and contained an embryo at the globular stage. A heart-shaped embryo had developed at 15 DAF in siliques 40–55 mm in length. A torpedo-shaped embryo was observed in siliques 55–65 mm in length at 31 DAF. The silique became green 10 days later by which time the embryo had entered the cotyledon stage. The cotyledonary-embryo stage lasted for 13 days until the silique became brown (Fig. 1A & B). The embryo diameter and seed size were measured from the globular to the cotyledonary stages (Fig. 1C). When the seed coat changed color from white to green, the embryo transited from the torpedo- to the early cotyledonary stage and a sharp increase in embryo diameter was detected without distinct change in seed size (Fig. 1A & C).

**Fig. 2:**
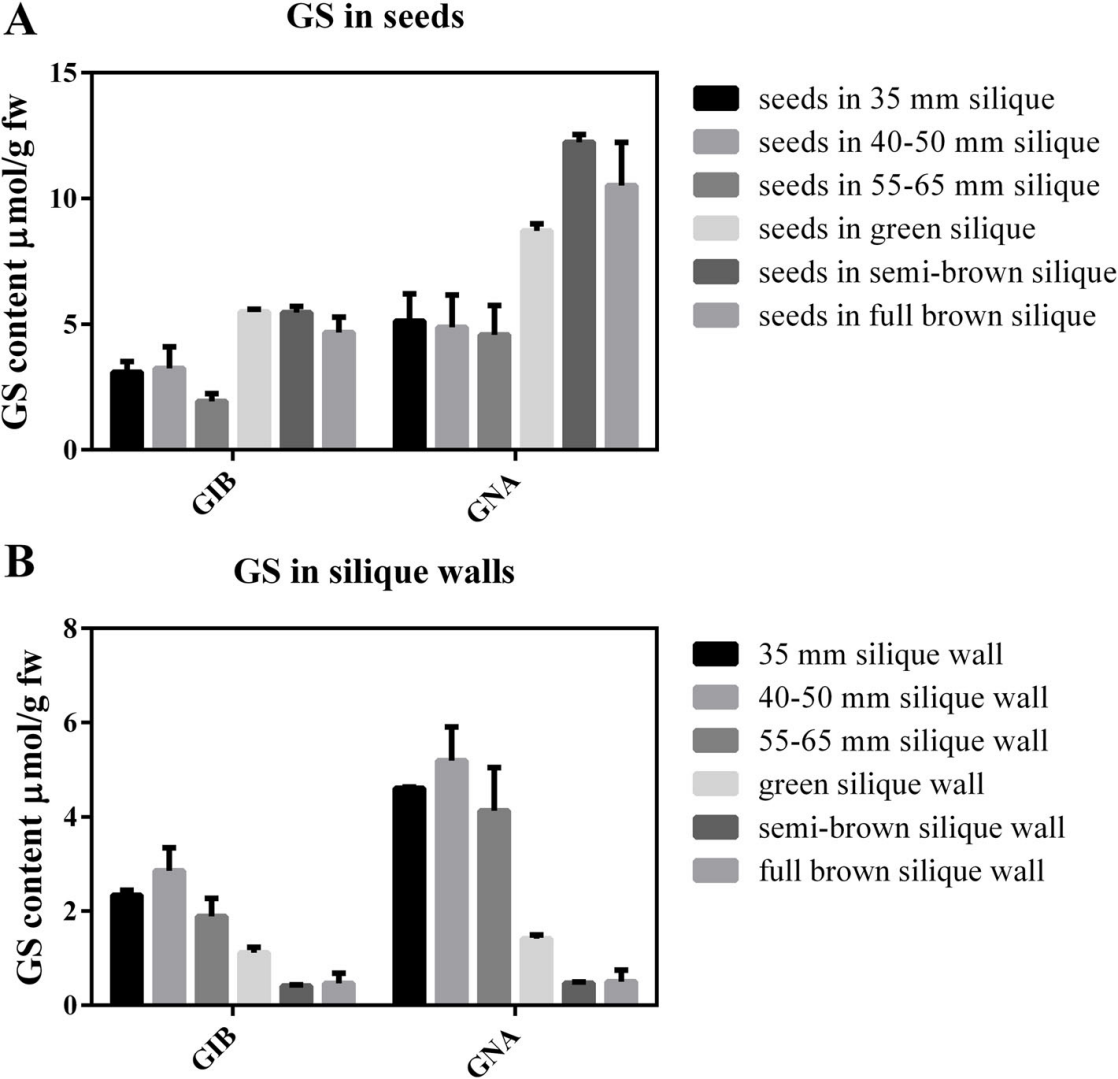
The content of predominant GS (GIB and GNA) in the seed (**A**) and corresponding silique wall (**B**) during the formation of Chinese kale seeds. The X axis represents the different profiles of GSs, GIB (Glucoiberin) and GNA (Gluconapin). The Y axis is the content of GSs. Error bars indicate ± SE

The GS content of the seed and corresponding silique walls was measured at different developmental stages (Fig. 2). Eight kinds of GSs were identified including four kinds of aliphatic GSs (glucoiberin, progoitrin, gluconapin, and glucoerucin) and four kinds of indolic GSs (glucobrassicin, 4-hydroxybrassicin, 4-methoxyglucobrassicin, and neoglucobrassicin) (Supplemental Table 1). Two aliphatic GSs, namely the 3-carbon glucoiberin (GIB) and 4-carbon gluconapin (GNA) predominated in the seed and silique wall (Supplemental Table 1). Accumulation of GIB and GNA in the seeds followed a similar pattern; contents were relatively low before the torpedo-embryo stage, increased when the embryo entered the cotyledonary stage, and thereafter high contents were maintained until the embryo matured (Fig. 2A). The change in GS content in the corresponding silique walls exhibited the opposite trend, i.e., GS content was high when the silique grew from 10 to 65 mm long, and low after the seed coat became green (Fig. 2B). It is worth noting that the salient change in GS content was observed from the torpedo-embryo stage to the early cotyledonary-embryo stage, and opposite trends for GS accumulation in the seed and corresponding silique wall were observed, indicating that GS may be transferred from the silique wall to the seed during embryo maturation.

### Analysis of transcriptome in seeds and silique walls at torpedo- and cotyledonary-embryo stages

To clarify the underlying mechanism of GS accumulation during seed formation, seeds and silique walls at the torpedo-embryo and the early cotyledonary-embryo stages were subjected to transcriptome sequencing. Twelve *transcriptome database*s, comprising seeds and silique walls at the torpedo-embryo stage (SC and PC, respectively) as well as seeds and silique walls at the early cotyledonary-embryo stage (SD and PD, respectively) with three replications of each group, were constructed.

A total of 58.15 million raw reads was obtained with an average of 6.47 Gb data for each sample. After filtering, 43.12 million clean reads were mapped to the *B. oleracea* genome. The mapped percentage ranged from 80.66 to 84.51%. The average Q30 of the clean reads was about 93% and ensured the high quality of sequence data and subsequent analysis (Table 1). We identified 49,281 genes, of which 43,580 were known genes and 5701 were novel genes. Among the 47,212 new transcripts detected, 22,070 were long-chain non-coding RNA, 19,321 were new alternative splicing isoforms of known protein-coding genes, and 5821 were transcripts of new protein-coding genes.

**Table 1:** Summary of RNA-Seq data sets

Sample	Total Raw Reads (M)	Total Clean Reads (M)	Mapped to genome (%)	Clean Reads Q30 (%)	Clean Reads Ratio (%)
**PC1**	57.15	42.94	82.77	93.14	**75.13**
**PC2**	58.78	43.10	82.77	92.89	**73.33**
**PC3**	57.15	43.14	81.18	93.27	**75.49**
**PD1**	58.31	42.65	81.21	92.76	**73.15**
**PD2**	58.78	43.53	82.17	92.83	**74.05**
**PD3**	57.98	43.45	81.11	92.86	**74.94**
**SC1**	59.62	43.36	81.08	92.89	**72.72**
**SC2**	59.72	43.45	81.25	92.86	**72.75**
**SC3**	59.60	43.39	80.66	92.91	**72.79**
**SD1**	59.62	43.09	82.70	92.53	**72.27**
**SD2**	55.52	42.78	83.59	93.58	**77.06**
**SD3**	55.52	42.50	84.51	93.48	**76.56**

**Fig. 3:**
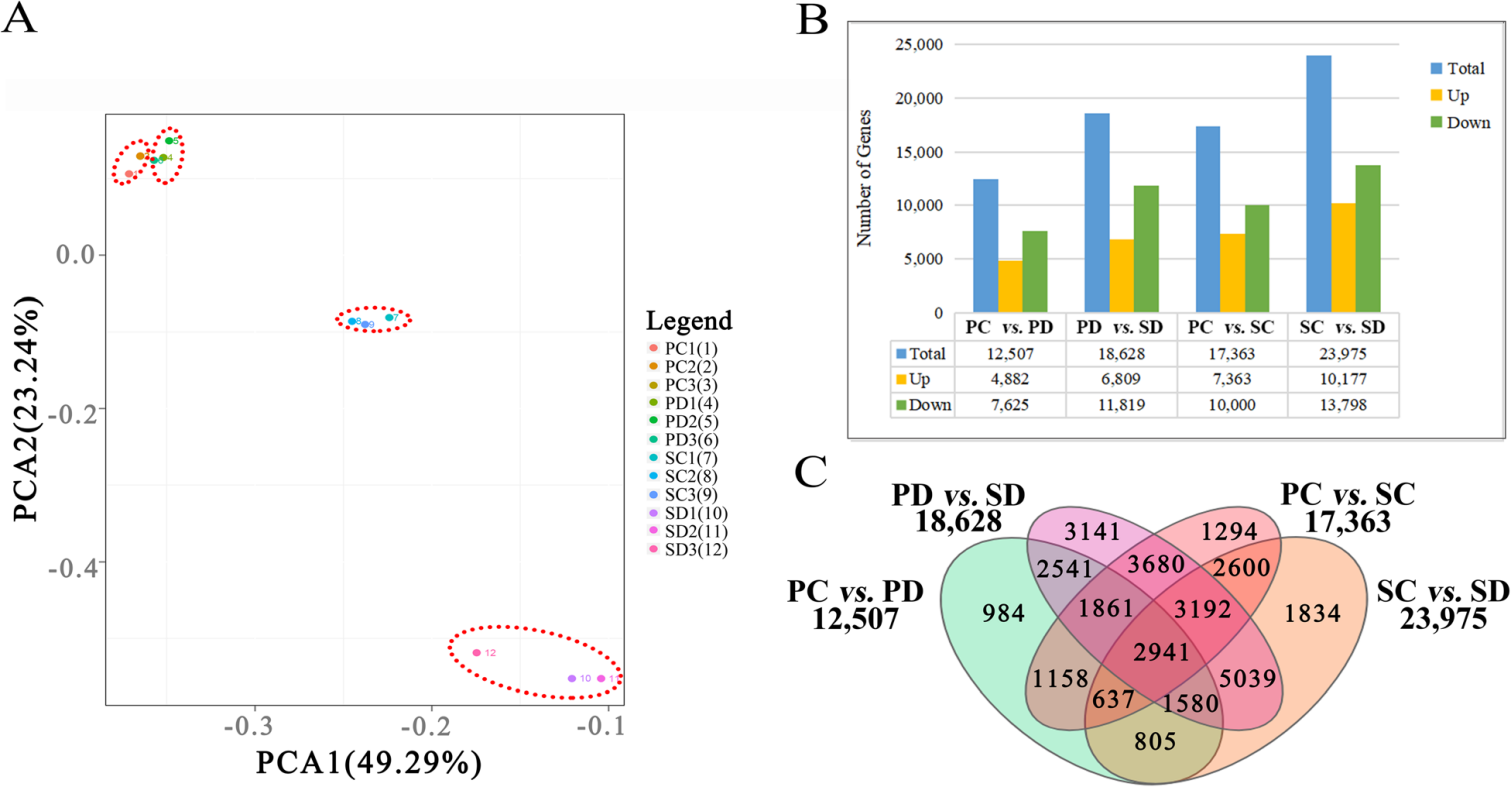
Qualification of RNA sequencing data in the seed and corresponding silique wall at the torpedo- and cotyledonary-embryo stages. **A** PCA analysis of different samples used for RNA sequencing. SC and PC means seed and silique wall at torpedo-embryo stage, SD and PD means seed and silique wall at cotyledonary-embryo stage. Each group contain three replicates. PC2 and PC3 have high similarity and overlap in the Figure. **B** Histogram of the numbers of differentially expressed genes (DEGs) identified in the seed and corresponding silique wall at torpedo- and cotyledonary-embryo stages. **C** Venn diagram showing the number of DEGs between differential groups in PC vs SC, PD vs SD, SC vs SD, and PD vs SD

Next, we analyzed the correlation of sequenced samples and the distribution of gene expression (Fig. 3). Principal component analysis (PCA) analysis was performed to evaluate the similarity of the 12 cDNA databases. The three replications of each of the four groups (SC, PC, SD, and PD) clustered together and gene expression was strongly correlated within each group (Fig. 3A). The differentially expressed genes (DEGs) in the four groups were counted in Fig. 3B. At the torpedo-embryo stage, 17,363 DEGs between SC and PC were detected, of which 7363 genes were up-regulated and 10,000 genes was down-regulated. At the cotyledonary-embryo stage, 23,975 DEGs between SD and PD were identified, of which 10,177 genes were up-regulated and 13,798 genes were down-regulated (Fig. 3B). During the transition in embryo development from the torpedo- to the cotyledonary-embryo stages, 12,507 and 18,628 genes were differentially expressed in the silique walls and seeds, respectively, among which 4882/6809 genes were up regulated and 7625/11,819 genes were down regulated (Fig. 3B). A total of 2941 DEGs common to the four groups were identified (Fig. 3C).

### Analysis of differentially expressed genes during seed formation

**Fig. 4:**
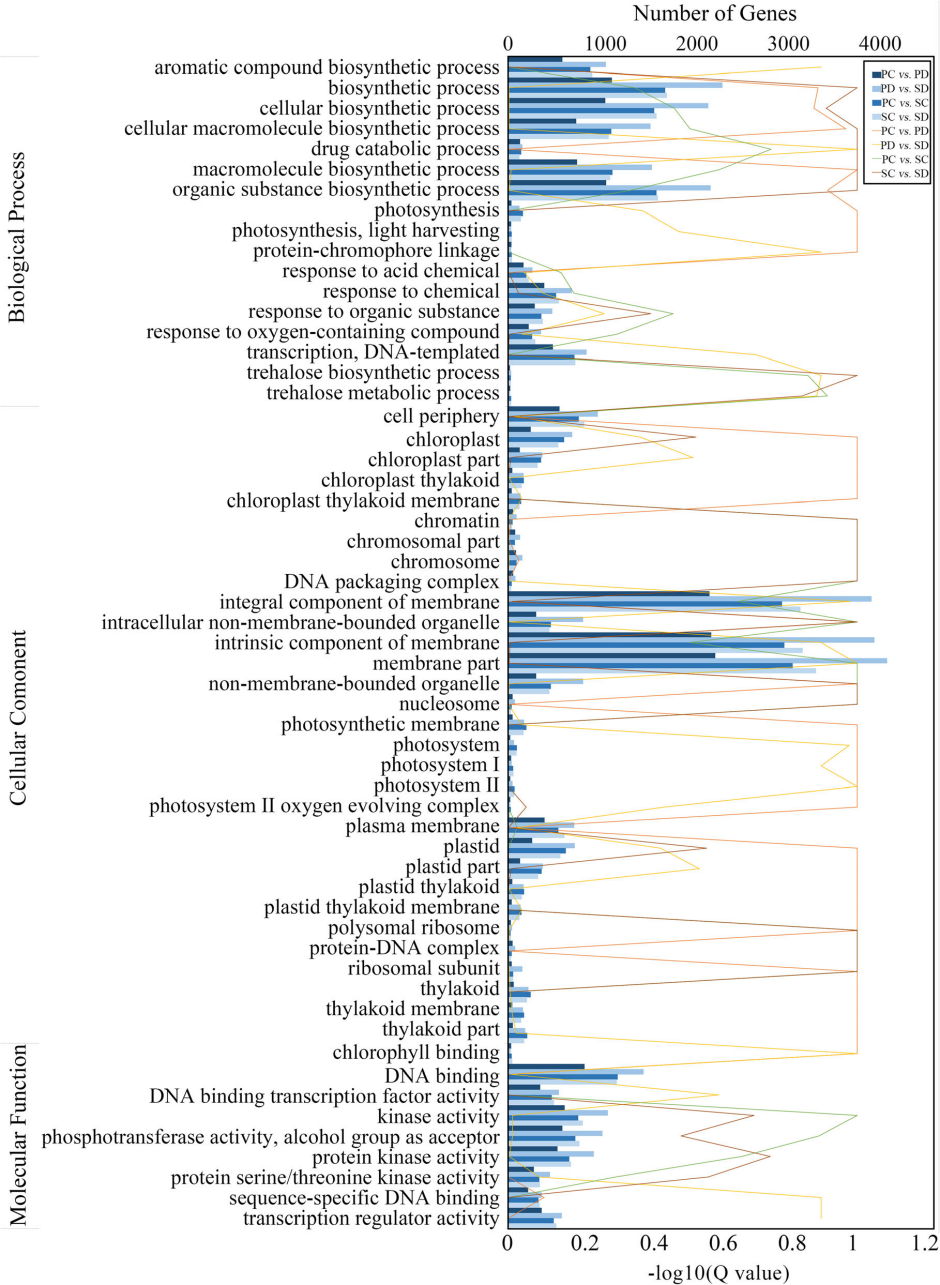
GO analysis of DEGs in PC vs PD, SC vs SD, PC vs SC, and PD vs SD. The upper X axis is the number of DEGs annotated to the GO Term, and the lower X axis represents the -log10 (Q-value). The Y axis is the GO term. Show the enrichment of GO Term in the form of a bar chart, and plotted the top 20 GO Term with the smallest Q-value

To identify the function of the DEGs, functional classification was performed based on annotations in the Gene Ontology (GO) database (Fig. 4, Supplemental Fig. 1). In total, 24,854 genes were assigned to the biological process (BP), cellular component (CC), and molecular function (MF) categories, which were enriched in 15, 23, and 11 terms, respectively (Supplemental Fig. 1). In SC vs SD, SC vs PC, PC vs PD, and SD vs PD comparisons, the DEGs exhibited a similar GO classification. The three most highly enriched subcategories of BP for all four groups were biosynthetic process, cellular biosynthetic process, and organic substance biosynthetic process. The most highly enriched CC subcategories were integral component of membrane, intrinsic component of membrane, and membrane part. The MF terms showing the highest enrichment were DNA binding, kinase activity, and phosphotransferase activity. In the BP, CC, and MF categories, abundant genes were classified into transcription regulation related processes (Fig. 4).

**Fig. 5:**
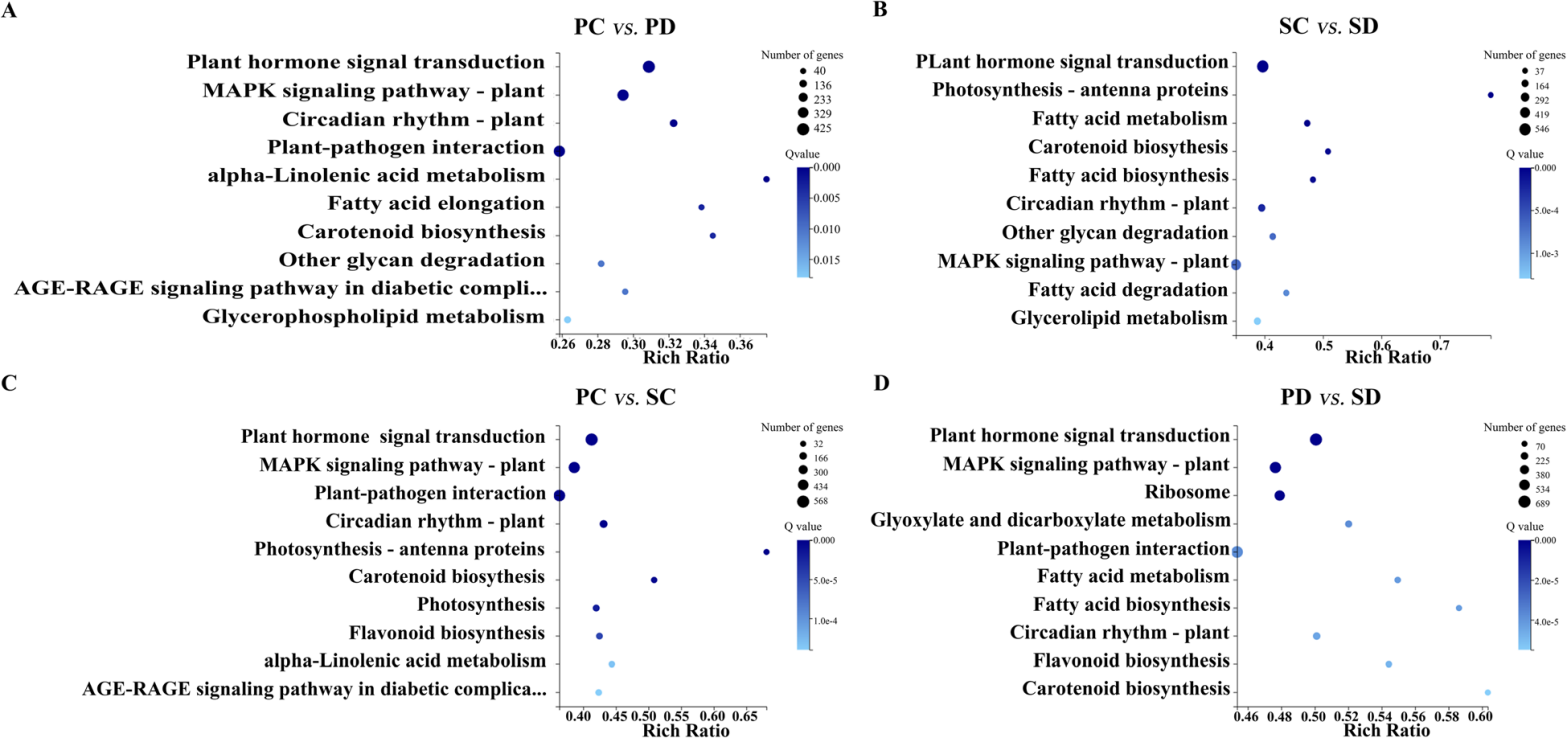
KEGG enrichment of DEGs in PC vs PD, SC vs SD, PC vs SC, and PD vs SD. The X axis is the Rich Ratio (Rich Ratio is calculated as candidate gene number in a specific term/ total gene numbers) and the Y axis represents KEGG pathway, the size of the bubble indicates the number of genes annotated to a KEGG Pathway. The blue color represents the enriched Q-value. The darker the color, the smaller the Q-value. Show the enrichment of KEGG pathway in the form of a bar chart, and plotted the top 10 KEGG pathway with the smallest Q-value

To explore the biological pathways in which the DEGs are involved, the Kyoto Gene and Genome Encyclopedia (KEGG) database was used for DEG classification [39, 40]. A total of 9637 DEGs were assigned to five branches with 19 subbranches (Supplemental Fig. 2). After enrichment analysis, the 10 top-ranked pathways with the highest gene numbers and a low Q value were screened and listed in Fig. 5. In PC vs PD, PC vs SC, SC vs SD, and PD vs SD comparisons, the pathway with the highest number of enriched DEGs was plant hormone signal transduction, followed by the MAPK signaling pathway.

### Expression pattern of GS biosynthesis and degradation associated genes during seed formation

**Fig. 6:**
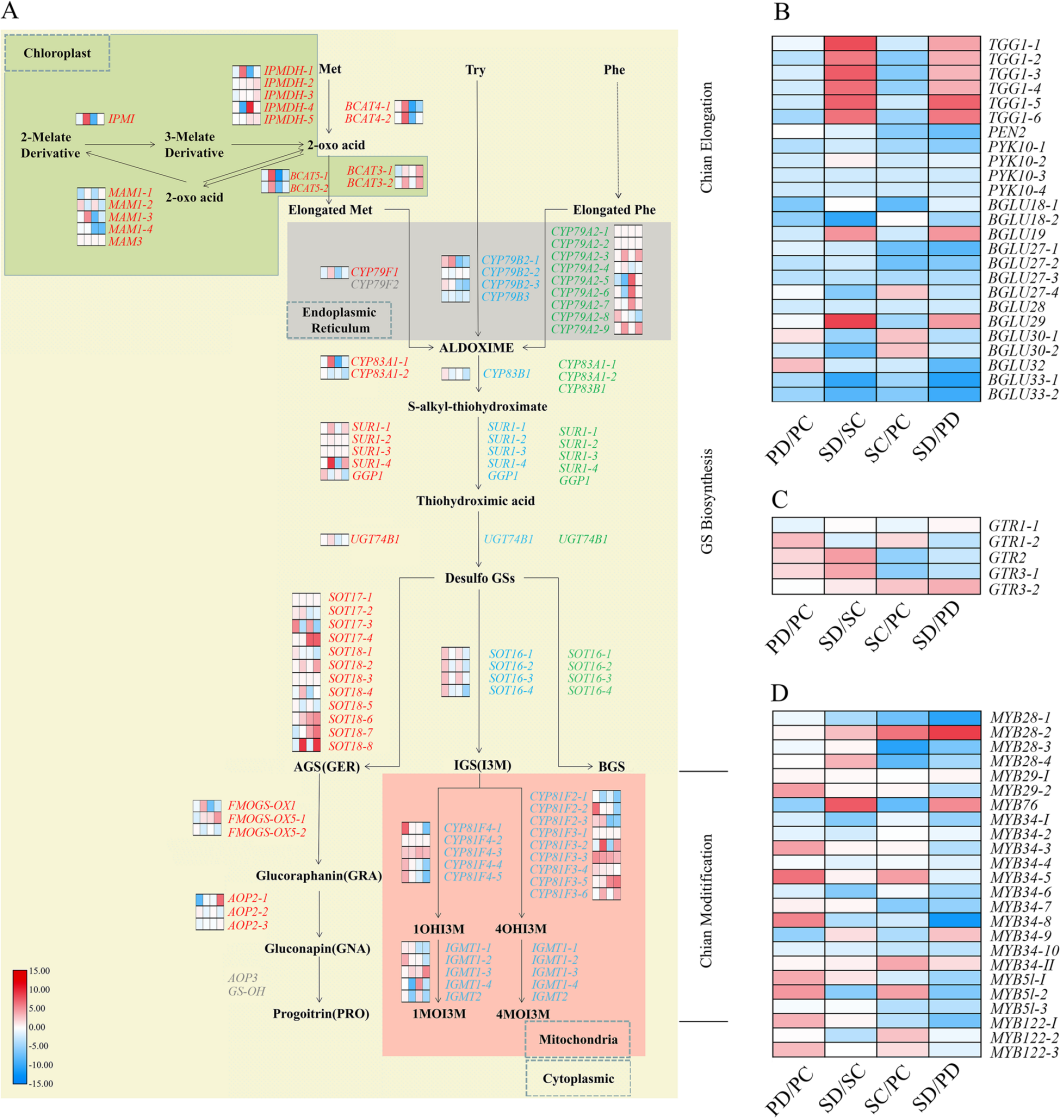
The diagram of GS biosynthesis and expression comparison of the genes related to GS biosynthesis and degradation in the seed and corresponding silique wall at the torpedo- and cotyledonary-embryo stages. **A** GS biosynthesis pathway in cells and the expression comparison of related genes in PC, PD, SC, and SD. The yellow part means cytoplasmic part in a cell, the green part represents chloroplast, the grey part represents endoplasmic reticulum, and the red part represents mitochondria. **B** Expression comparison of myrosinase encoding genes in PC, PD, SC, and SD. **C** Expression comparison of GS transporter genes *GTRs* in PC, PD, SC, and SD. **D** Expression comparison of six transcriptional factors including *MYB28*, *MYB29*, *MYB76*, *MYB34*, *MYB51*, and *MYB122* in PC, PD, SC, and SD. The heatmap from left to right is the comparison between PD and PC, SD and SC, SC and PC, as well as SD and PD, respectively

The biosynthesis of GS involves three independent stages: elongation of aliphatic GS precursors, formation of the core structure and modification of side chains (Fig. 6A, Supplemental Table 2).

Seventeen genes associated with elongation of aliphatic GS precursors were identified in the transcriptome of the seed and corresponding silique wall of Chinese kale. These genes consisted of two branched chain aminotransferase 4 (*BCAT4*), two bile acid transporter 5 (*BAT5*) genes, four methylthioalkyl malate synthase 1 (*MAM1*) genes, one methylsulfidealkenyl malate synthase 3 (*MAM3*), one isopropylmalate isomerase (*IPMI*), five isopropylmalate dehydrogenases (*IPMDH*) genes and two branched chain aminotransferase 3 (*BCAT3*) genes. At the torpedo- and cotyledonary-embryo stages, the expression of *BCAT4–1*, *BAT5–1*, *MAM1–3*, and *MAM1–4* was lower in the seed than in the corresponding silique wall. In comparisons of the seed and silique wall, at the torpedo-embryo stage higher transcript levels for two *BCAT4* genes, two *BAT5*s genes, *MAM1–3*, *IPMI*, *IPMDH1–1*, and *BCAT3–2* were detected in the seed, whereas fewer transcripts of *MAM1–1* and *BCAT3–1* were detected in the silique wall at the cotyledonary-embryo stage (Fig. 6A).

With regards to formation of the core structure, 12 genes comprising 39 members were mapped from the databases. These genes consisted of the cytochrome *P450* homologs *CYP79A2*, *CYP79B2*, *CYP79B3*, *CYP79F1*, *CYP83A1*, and *CYP83B1*, carbon-sulfur lyase (*SUR1*), γ-glutamyl polypeptide synthetase (*GGP1*), glucosyltransferase (*UGT74B1*), and desulfurized GS transferase genes *SOT16*, *SOT17*, and *SOT18*. In the synthesis of core structure of aliphatic GS, one *CYP79F1*, two *CYP83A1*, four *SUR1*, one *GGP1*, one *UGT74B1*, four *SOT17*, and eight *SOT18* genes were identified. In the seed, the expression of *CYP79F1*, *CYP83A1–1*, *CYP83A1–2*, *UGT74B1*, *SOT17–2*, *SOT17–4*, *SOT18–1*, *SOT18–4*, and *SOT18–5* was down regulated compared with that in the corresponding silique wall. Among genes with multiple members, such as *SOT17* and *SOT18*, some members (*SOT18–6* and *SOT18–7*) showed an increased expression level in the seed compared with that in the silique wall, whereas other members (*SOT17–3* and *SOT18–8*) showed the opposite trend in SC vs PC and SD vs PD comparisons. In the comparison SD vs SC, the expression of *CYP83A1–1*, *SOT18–4*, *SOT18–6*, and *SOT18–8* was higher in SD, whereas expression of *CYP83A1–2* and *SOT18–5* was lower in SD, compared with that in SC (Fig. 6A). For genes related to the synthesis of indolic and aromatic GS core structure, nine *CYP79A2*, three *CYP79B2*, one *CYP79B3*, one *CYP83B1*, and four *SOT16* genes were identified. The expression of these genes is highlighted in Fig. 6A in blue and green, respectively.

At the last step of secondary modification, six genes associated with aliphatic GS biosynthesis were identified including three monooxygenases (*FMOGS*) comprising one *FMOGS-OX1*, two *FMOGS-OX5* genes, and three 2-oxoglutarate-dependent dioxygenases (*AOP2*) *genes.* Similar to the afore mentioned expression patterns of multigene families, *FMOGS* gene members also showed differential expression patterns. The expression level of *FMOGS-OX1* was decreased, whereas the expression of *FMOGS-OX5–1* was increased in the comparison of PC vs SC and PD vs SD. The expression of *FMOGS-OX5–2* was higher in PC, PD, and SC, whereas lower in SD (Fig. 6A). Genes involved in modification of indolic GS were also detected, including 14 *CYP84F* and five *IGMT* genes. The proteins encoded by these genes were predicted to be localized in mitochondria and their expression level varied in different samples (Fig. 6A).

With regards to degradation of GS, six typical myrosinases (*TGG1*) were identified in the sequence database (Fig. 6B, Supplemental Table 2). The expression of *TGG1* genes was relatively consistent in the developing seeds and silique walls. At the torpedo-embryo stage, transcript levels for *TGG1–2*, *TGG1–3*, and *TGG1–6* genes were lower in SC compared with PC, whereas those of *TGG1–1*, *TGG1–4*, and *TGG1–5* showed no obvious difference between SC and PC. At the early cotyledonary-embryo stage, abundant transcripts of *TGG1* genes in the seed were detected and the expression of all *TGG* genes was significantly up regulated in the seed compared with the corresponding silique wall, and transcript levels were markedly higher than in the seed at the torpedo-embryo stages. Thus, it is proposed that during the transition of the embryo from the torpedo- to the early cotyledonary stage, myrosinases encoded by typical *TGG1* genes accumulated massively in the seeds. With regards to specific degradation of indolic GS, expression of *PEN2* was lower in the seed compared with the silique wall and remained stable with embryo development (Fig. 6B). Other β-thioglucosidases that showed myrosinase activity were identified and the differential expression of these genes was also summarized in Fig. 6B.

Five *GTR* genes including two *GTR1* genes, one *GTR2*, and two *GTR3* genes, were annotated in the transcriptome (Fig. 6C, Supplemental Table 3). Higher expression of *GTR2* was detected in PC (vs SC) and PD (vs SD), which indicated that aliphatic GS may be transferred from the silique wall to the seed. Compared with the seed at the torpedo-embryo stage, GS transportation to the seed may be enhanced at the early cotyledonary-embryo stage because *GTR2* transcript level was significantly enhanced in SD compared with that of SC. The transcript level of *GTR1–2* was increased in PD (vs SD), but not in PC (vs SC). This difference may be related to the enhancement of *GTR1–2* in the silique wall at the early cotyledonary-embryo stage as higher abundance of *GTR1–2* transcripts was detected in PD than in PC. The gene *GTR1–1* showed no obvious difference in transcript abundance among all four groups. The two *GTR3* members showed opposite expression patterns. In SC vs PC and SD vs PD comparisons, expression of *GTR3–1* was decreased, whereas expression of *GTR3–2* was increased, respectively.

### Identification of transcriptional factors regulating GS biosynthesis

Positive transcription regulators of the *MYB* family were annotated in the transcriptome (Fig. 6D, Supplemental Table 4). In *Arabidopsis*, *MYB28*, *MYB29*, and *MYB76* are transcriptional factors regulating aliphatic GS biosynthesis, and *MYB34*, *MYB122*, and *MYB51* are involved in the regulation of the synthesis of indolic GS [41]. In the present study, with regards to regulation of aliphatic GS, four *MYB28*s, two *MYB29*s, and one *MYB76* gene were identified. At the torpedo- and cotyledonary-embryo stages, expression of *MYB28–1*, *MYB28–3*, and *MYB28–4* was down regulated, whereas *MYB28–2* was up-regulated in the seed compared with the corresponding silique wall. Expression of *MYB76* differed in all four groups. In SC vs PC at the torpedo-embryo stage, fewer *MYB76* transcripts were detected in the seed. At the early cotyledonary-embryo stage, the *MTB76* transcript level was significantly increased in the seed but decreased in the silique wall compared with those at the torpedo-embryo stage, and resulted in greater abundance of *MYB76* transcripts in SD compared with that of PD. The expression of *MYB29* was high in the silique wall at the cotyledonary-embryo stage and higher than that detected in the corresponding seed and in the silique wall at the torpedo-embryo stage. With respect to the regulation of indolic GS, the largest gene family was *MYB34*, which comprised 11 members. Besides, three *MYB51* and three *MYB122* genes were identified, of which the majority were more highly expressed in the silique wall than in the seed (Fig. 6D).

**Fig. 7:**
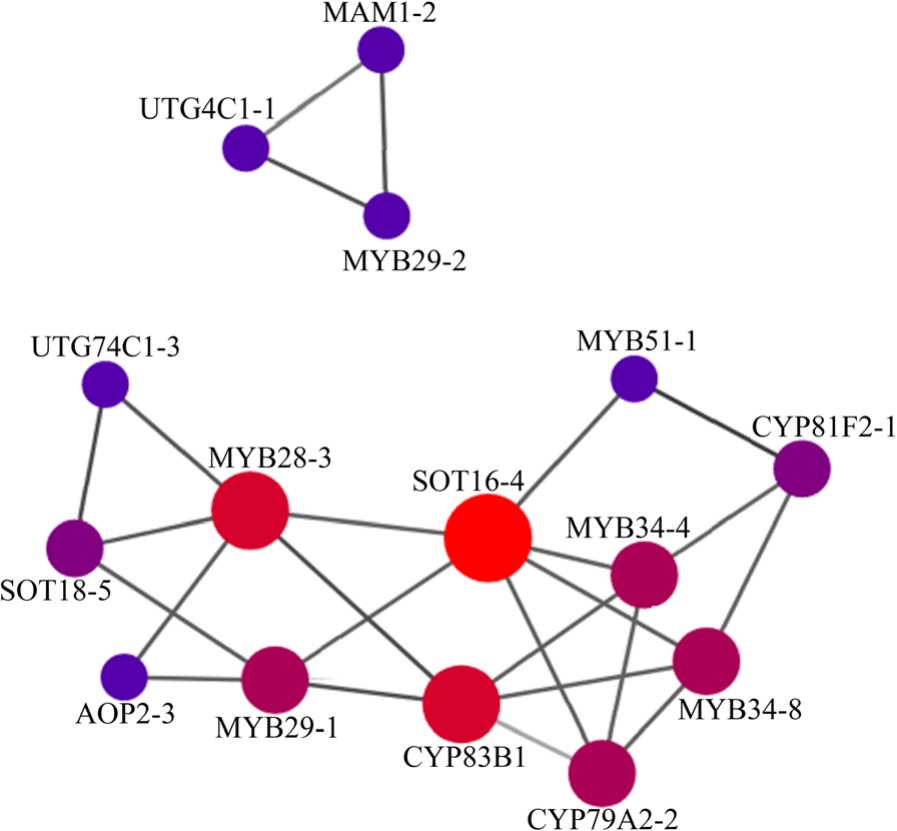
Protein interaction network of GS regulatory factors and its biosynthetic genes. Each point in the figure represents a protein and the line indicates that an interaction between the two proteins

To explore the regulation of GS metabolism, the possible interaction of GS metabolism-related genes was tested by means of protein-protein interaction assays. Only some transcription factor gene members were involved in protein-protein interaction (Fig. 7, Supplemental Table 4). Among 24 identified MYBs, six MYBs including MYB28–3, MYB29–1, MYB29–2, MYB34–4, MYB34–8, and MYB51–1 were predicted to regulate the synthetic protein. In the regulation of aliphatic GS, MYB28–3 showed close affinity for AOP2–3 and SOT18–5. Surprisingly, aliphatic GS-related MYB28–3 interacted with the indolic GS synthetic protein SOT16–4 and CYP83B1, which also could be regulated by MYB34, indicating a possible transcription factor-mediated crosstalk between indolic and aliphatic GSs.

## Discussion

To understand the mechanism of GS accumulation in Chinese kale seeds, we first analyzed the accumulation pattern of GS in the seed and corresponding silique walls during embryo development. Two crucial consecutive stages (torpedo-embryo and early cotyledonary-embryo stages) were selected based on their differential GS content, and genes related to GS metabolism were mapped to the genome. In total, 135 genes were identified, of which 24 genes were transcription factors, 81 genes were associated with biosynthetic pathways, 25 genes encoded catabolic enzymes, and five genes matched with transporters. The expression of these genes in the two selected stages were analyzed. Significant change in GS accumulation in the seed occurred in the transition from the torpedo-embryo stage to the early cotyledonary-embryo stage. Gene analysis confirmed the transportation of GSs from silique walls to seeds during the transition from torpedo- to early cotyledonary-embryo stage. Moreover, the high expression of sidechain modification related genes *FMOGS* and *AOP2* indicates possible modification of glucosinolate exist in seeds.

### Accumulation of GS in Chinese kale seeds

In *Arabidopsis*, the silique wall is considered to be the predominant source of GS in the seed [42]. In Chinese kale, GS in the silique wall also plays an important role in GS accumulation in the seed. Although greater quantities of GS accumulated in the seed than in the silique wall (Fig. 2), the expression level for the majority GS biosynthetic genes was lower in the seed than in the corresponding silique wall (Fig. 6A). This discrepancy between gene expression and GS accumulation may be attributed to the large amounts of *GTR* genes expressed in the silique wall compared with those in the seed. It has been proven that GTRs are responsible for transportation of GS from the silique wall to seed [32]. In the present study, with development of the embryo, the expression levels of *GTR*s were enhanced, which indicated that an increasing quantity of GS needed to be transported from the si1lique to the seed.

Transportation and synthesis of GS are important sources for the accumulation of GS. Previous studies have investigated factors underlying the abundance of GS in the seed, especially whether the seed is capable of synthesizing GS [31, 43]. The findings that support an absence of GS synthesis in the seed are i) maternal control of seed GS content, as the F1 progeny always exhibit similar GS profile to that of the female parent in reciprocal crosses [44]. ii) in silico microarray expression of the key GS chain elongation gene *MAM1* and the core structure gene *CYP79F1* is too low to affect GS accumulation in the seed [31]. Recently, the mutation of transporter genes *GTR* in Arabidopsis confirmed no GS biosynthesis in seeds since the *gtr* double mutant showed no GS accumulation in seeds [32].

In the present study, almost all homologous genes of GS biosynthetic genes were identified in Chinese kale seeds and silique walls. *CYP79F1* and *MAM1* were two of these genes and both showed much higher transcript levels in the silique wall than in the seed at the torpedo- and cotyledonary embryo stages. Moreover, the high expression of sidechain modification of GS related genes was noted in seeds at the torpedo-embryo stage, especially *FMO* and *AOP2* genes. In the biosynthesis of glucosinolate, Chinese kale ‘Huanghua’ in the present study characterized the production of alkenylated GS, GNA, which is dependent on the proper expression of side chain modification related gene *AOP2*. With GNA as the main GS in plant, it is Arabidopsis Cvi ecotype. However, research taking Cvi as material in GS accumulation is limited and mostly used one is Col-0, which cannot produce alkenyl GS because of 5-bp loss in *AOP2* gene [45–47]. Similar to the GS change in varied Arabidopsis ecotypes, the Brassica vegetables also accumulate different kinds of glucosinolates depending on the differential expression of *AOP2*. Three alleles of *AOP2* were identified in *B. oleracea*, two of which have no function due to the generation of premature stop codons [48]. In kale (*B. oleracea* var. *viridis*), the expression of *BoAOP2* could catalyze the formation of alkenyl GSs from methylsulfinyl butyl GS (glucoraphanin, GRA) [49]. In broccoli (*B. oleracea var. italica*), there is 2 bp deletion in the exon of *AOP2* gene, which results in the malfunction of AOP2 and the broccoli mainly accumulates GRA [50]. In cauliflower (*B. oleracea var. botrytis*) and cabbage (*B. oleracea var. capitata*), the main GSs are sinigrin (2-propenyl GS) and/or PRO depending on different varieties used [51]. In *B. rapa*, three *AOP2* homologs were aligned to the *AtAOP2*. The dominant glucosinolates in *B. rapa* is GNA, glucobrassicanapin (4-Pentenyl GS), and PRO [52]. Thus, we proposed that the secondary modification of aliphatic GS by expression of *AOP2* might be related to the accumulation of GNA in Chinese kale during the formation of seeds. Also, we cannot exclude the possibility that the amount of GNA in the silique wall or other sources is enough for the seed accumulation of GNA. Further experiments are still needed to verify the function of AOP2 in GS accumulation during seed formation.

### Regulation of GS accumulation in Chinese kale

In contrast to *Arabidopsis*, multiple members (ranging from 1 to 9) of GS metabolism-related genes were identified in Chinese kale. Gene redundancy causes difficulties for the management of GS content and profiles by gene editing, which requires precise gene targets [53, 54]. Transcriptome analysis reveals the expression of all gene members in one family, and genes that are not expressed can be excluded. For example, *AOP2* is critical for alkenylation of its substrates and the product GNA is the predominant GS accumulated in Chinese kale. To regulate the GNA content in Chinese kale seeds, *AOP2* with a high expression level may be the target. However, three *AOP2* gene members were detected in Chinese kale, which complicates selection of the correct AOP2 to target. From the present analysis, the priority should be given to *AOP2–2* as other members showed no distinct changes at the transcriptional level. Protein-protein interaction assays are also needed to examine the possible relationships among proteins, which can help to reduce the complexity caused by multiple gene members. For example, the aliphatic GS transcription factor *MYB28* consisted of four members in Chinese kale but only MYB28–3 was predicted to interact with SOT18–5 and AOP2–3.

Accompanying GS accumulation during seed formation, abundant *TGG* transcripts were detected in the seed (Fig. 6B). Interestingly, the enhancement of *TGG* transcript level in the seed occurred during the transition from the torpedo-embryo stage to the early cotyledonary-embryo stage. At this transition, expression of the most typical *TGG* member, *TGG1*, decreased in SC (vs PC) and increased in SD (vs PD), which indicated that *TGG1* may play an important role in GS accumulation. The turnover of GS provides building blocks (e.g., glucose, sulfate, sulfur, ammonia, and carboxylic acid) for primary metabolism, especially during germination [10, 55]. Recently, an increasing number of atypical TGGs have been observed to be involved in GS degradation [30]. Different degradative enzymes are indicated to be required for different biological processes. Meier et al. (2019) revealed that functional nitrile-specifier proteins (NSP) are necessary for GS degradation during germination from days 4 to 10 by analysis of the change in GS content in *nsp* mutant lines [55]. During Chinese kale seed development, the high expression level of *BGLU29* in the seed at the early cotyledonary stage was also noted. Accumulation of myrosinase during seed development not only provides a physical protective barrier for seed formation, but may also play a role as a potential sulfur provider during germination. However, the specific function of *TGG* in seeds remains elusive. Direct participation of typical and atypical *TGG* in GS degradation at different stages of seed development requires further investigation.

## Conclusions

The torpedo-embryo stage and the early cotyledonary-embryo stage were identified as critical in GS accumulation during Chinese kale seed development. The expression of genes related to GS metabolism in these two stages was analyzed and the transportation of GS from the silique wall to the seed was confirmed in the transition of seeds from torpedo-embryo stage and the early cotyledonary-embryo stage. In addition, the high expression of AOP2 in seeds at the early cotyledonary-embryo stage indicate possible sidechain modification would happen during seed formation.

## Methods

### Plant materials

Chinese kale (*B. oleracea c*v HuangHua) seeds were purchased from Gaoda seed shop (Fuzhou, China) and used for the field experiments (Fujian Agriculture and Forestry University). The seeds were sprinkled evenly in a petri dish (diameter = 15 cm) with moist perlite, and placed in a 28 °C chamber (16 h light/8 h dark photoperiod) until the cotyledons were fully expanded. Then the seedlings were transferred to a culture medium containing peat soil: vermiculite: perlite at 3:1:1 and placed in an artificial climate chamber (MGC-450HP-2, Shanghai Yiheng Technology Co., Ltd.) at 28 °C with 16 h light/8 h dark photoperiod. After 1 month of cultivation, the kale seedlings were planted in the field, the row distance × row spacing was 25 cm × 30 cm, and the protective rows were set around the field. The field trials were conducted in three consecutive years (201609–201704, 201709–201804, and 201809–201904) with three repetitions which contain at least 24 plants each time. The soil was deeply ploughed and tilled to ensure that the soil conditions and other field management procedures were equal for all the accessions evaluated in this trial. Field management such as daily watering and fertilization was performed regularly until the plants enter the reproductive stage.

After the flower buds emerged, the branches were labelled and development of the seeds was recorded. When the flowers were fully expanded, the stage of embryo in seeds was observed under a microscope (Olympus IX73, Japan). The embryo developed from the globular stage to the cotyledonary stage. The seeds were divided into different stages based on the development of embryo in seeds. The seeds and corresponding silique walls were harvested and classified. Then samples were quickly frozen in the liquid nitrogen and stored in a − 80 °C refrigerator for the following measurements.

### Measurement of GS content during the formation of seeds

The determination and analysis of GS were carried out with reference to the test method of Guo et al. (2016) and optimized [3]. 200 mg sample was added to 2 mL of boiled ddH_2_O at 100 °C for 10 min, the supernatant was then collected and the above operation was repeated. The supernatant combined twice were used as crude extract, and then added 30 mg of activated DEAE-Sephadex to the purification column. Washed with 0.02 mol/L pyridine acetate, ddH_2_O, then added 1 mL of crude extracts to the column. Then washed again with pyridine acetate and ddH_2_O. 100 μL of 0.1% Sulfatase was added for 14 h, and then eluted with water to obtain desulfurized GS. The analysis of GS was performed by ultra-high-phase liquid chromatography (UPLC), using Water’s TUV detector (Waters, Milford, USA). An UPLC BEH C18 (1.7 μm particle size, 2.1 mm × 50 mm, Waters, Milford, USA) was used with a mobile phase of acetonitrile and water. The analytical conditions were: flow rate at 0.4 mL/min, detection wavelength at 226 nm, injection volume 10 μL.

### Total RNA extraction and library construction

The RNA of seeds and silique walls were extracted using Trizol reagent (Invitrogen, Carlsbad, CA, USA) from Chinese kale in two stages and repeated three times, respectively. The purity of the samples was measured using a NanoDrop 1000 spectrophotometer (Thermo Fisher Scientific, Wilmington, DE, USA) and the concentration of the RNA samples was measured using a Qubit® 2.0 fluorometer (Life Technologies, CA, USA). Twelve independent transcriptome databases were analyzed using RNA sequencing, with an average insertion length of 200 bp for each of the twelve transcriptome databases, and data was synthesized using a genomic sample kit (Illumina, San Diego, CA). The concentration and size of the database were measured on a bioanalyzer using Agilent 2100 kit (Agilent, Palo Alto, CA). High-throughput sequencing was performed via an Illumina HiSeq 2500 instrument (MGI Tech Co., Ltd., China) with a read length of PE125.

### Sequencing data processing and analysis

Based on Sequencing by Synthesis technology, the qualified databases were sequenced by Illumina high-throughput sequencing platform (BGI sequencing, Shenzhen, China). The raw data was filtered to remove low-quality reads, linker contamination and high levels of unknown base N content. This project used SOAPnuke for statistics [56] and trimmomatic for filtering [57]. Bowtie2 was then used to compare clean reads to the reference genome (http://plants.ensembl.org/Brassica_oleracea/Info/Index) [58], and then RSEM was used to calculate the transcript expression level of genes [59]. All the sequences were aligned with NR, GO, KEGG databases to identify their function [39, 60–62].

### Identification of differentially expressed genes in the seed and corresponding silique wall at different stages

DEGseq was used for differential expression analysis between sample groups [63], and FPKM was used to analyze the expression level of differential genes, and the Benjamini-Hochberg method was used to correct the significant *P* values. Finally, the corrected P value, that is, Q value ≤0.001, |log2(fold change) | ≥ 2 as a screening criterion for the significance of differentially expressed genes, and Q value ≤0.001, |log2(fold change) | ≥ 4 as a screening criterion for the extremely significant difference of differentially expressed genes.

### Statistics analysis

PCA in Fig. 3A was analyzed by using the princomp function in R software and drawn by using the ggplot2 package in R software [64]. Heatmap in Fig. 6 was obtained by using TBTools [65]. DIAMOND (https://github.com/bbuchfink/diamond) was used to compare genes to the STRING database [66] for analysis of interaction among proteins in Fig. 7.

## Supplementary Information

**Additional file 1: Supplemental Fig. 1.** GO classification of differential expressed genes (DEGs). The X axis represents the number of genes annotated to the GO Terms, and the Y axis represents the classification of GO.

**Additional file 2 Supplemental Fig. 2.** KEGG classification of DEGs. The X axis is the number of genes ann:otated to a KEGG pathway category, and the Y axis is the KEGG pathway category.

**Additional file 3: Supplemental Table 1.** Glucosinolate profiles and their content in the seed and corresponding silique walls at different stages.

**Additional file 4: Supplemental Table 2.** GS biosynthesis and degradation related genes in Chinese kale. The value of log2 (B/A) reflects the comparison of gene expression level in B compared with the A, greater than 0 means that the gene expression level of the treatment group is up-regulated, and less than 0 means that it is down-regulated. A represents the control group, B represents the treatment group. When log2(B/A) > 2, it means significantly regulated.

**Additional file 5: Supplemental Table 3.** GS transporter related genes in Chinese kale. The value of log2 (B/A) reflects the comparison of gene expression level in B compared with the A, greater than 0 means that the gene expression level of the treatment group is up-regulated, and less than 0 means that it is down-regulated. A represents the control group, B represents the treatment group. When log2(B/A) > 2, it means significantly regulated.

**Additional file 6: Supplemental Table 4.** MYB transcriptional factors related genes to GS biosynthesis in Chinese kale. The value of log2 (B/A) reflects the comparison of gene expression level in B compared with the A, greater than 0 means that the gene expression level of the treatment group is up-regulated, and less than 0 means that it is down-regulated. A represents the control group, B represents the treatment group. When log2(B/A) > 2, it means significantly regulated.

## Data Availability

The materials of this study were provided by College of Horticulture at Fujian Agriculture and Forestry University. Correspondence and requests for materials should be addressed to Rongfang Guo (guorofa@163.com).

## Abbreviations

AOP: Oxoglutarate-dependent dioxygenases
BCAT: Branched chain aminotransferase
BGLU: β-Thioglucosidases
BP: Biological process
CC: Cellular component
DAF: Days after flowering
DEG: Differentially expressed gene
FMOGS: Monooxygenases
GGP: γ-Glutamyl polypeptide
GIB: Glucoiberin
GNA: Gluconapin
GO: Gene ontology
GRA: Glucoraphanin
GS: Glucosinolate
IPMI: Isopropylmalate isomerase
IPMDH: Isopropylmalate dehydrogenases
ITC: Isothiocyanate
KEGG: Kyoto gene and genome encyclopedia
MAM: Methylthioalkyl malate synthase
MF: Molecular function
NRT1/PTR: Nitrate transporter 1/peptide transporter
NSP: Nitrile-specifier proteins
PC: Silique walls at the torpedo-embryo stage
PCA: Principal component analysis
PEN2: Penetration 2
SC: Seeds at the torpedo-embryo stage
SD: Seeds at the early cotyledonary-embryo stage
PD: Silique walls at the early cotyledonary-embryo stage
TGG: Thioglucoside glucohydrolase
UPLC: Ultra-high-phase liquid chromatography

## References

[1] Lei J, Chen G, Chen C, Cao B (2017). Germplasm diversity of Chinese kale in China. Horticultural Plant J.

[2] Guo R, Deng Y, Huang Z, Chen X, XuHan X, Lai Z (2016). Identification of miRNAs affecting the establishment of Brassica Alboglabra seedling. Front Plant Sci.

[3] Guo R, Huang Z, Deng Y, Chen X, XuHan X, Lai Z (2016). Comparative transcriptome analyses reveal a special glucosinolate metabolism mechanism in Brassica alboglabra sprouts. Front Plant Sci.

[4] Chen J, Chen Z, Li Z, Zhao Y, Chen X, Wang-Pruski G, Guo R (2021). Effect of photoperiod on Chinese kale (Brassica alboglabra) sprouts under white or combined red and blue light. Front Plant Sci.

[5] Guo R, Shen W, Qian H, Zhang M, Liu L, Wang Q (2013). Jasmonic acid and glucose synergistically modulate the accumulation of glucosinolates in Arabidopsis thaliana. J Exp Bot.

[6] Wittstock U, Burow M (2010). Glucosinolate breakdown in Arabidopsis: mechanism, regulation and biological significance. Arabidopsis Book Am Soc Plant Biol.

[7] Wu X, Huang H, Childs H, Wu Y, Yu L, Pehrsson PR (2021). Glucosinolates in Brassica vegetables: characterization and factors that influence distribution, content, and intake. Annu Rev Food Sci Technol.

[8] Pfalz M, Mikkelsen MD, Bednarek P, Olsen CE, Halkier BA, Kroymann J (2011). Metabolic engineering in Nicotiana benthamiana reveals key enzyme functions in Arabidopsis indole glucosinolate modification. Plant Cell.

[9] Jeon J, Bong SJ, Park JS, Park Y-K, Arasu MV, Al-Dhabi NA, Park SU (2017). De novo transcriptome analysis and glucosinolate profiling in watercress (Nasturtium officinale R. Br.). BMC Genomics.

[10] Kissen R, Rossiter JT, Bones AM (2009). The ‘mustard oil bomb’: not so easy to assemble?! Localization, expression and distribution of the components of the myrosinase enzyme system. Phytochem Rev.

[11] Bhat R, Vyas D (2019). Myrosinase: insights on structural, catalytic, regulatory, and environmental interactions. Crit Rev Biotechnol.

[12] Wittstock U, Gershenzon J (2002). Constitutive plant toxins and their role in defense against herbivores and pathogens. Curr Opin Plant Biol.

[13] Bednarek P, Piślewska-Bednarek M, Svatoš A, Schneider B, Doubský J, Mansurova M, Humphry M, Consonni C, Panstruga R, Sanchez-Vallet A (2009). A glucosinolate metabolism pathway in living plant cells mediates broad-spectrum antifungal defense. Science.

[14] Bejai S, Fridborg I, Ekbom B (2012). Varied response of Spodoptera littoralis against Arabidopsis thaliana with metabolically engineered glucosinolate profiles. Plant Physiol Biochem.

[15] Guo R, Wang X, Han X, Li W, Liu T, Chen B, Chen X, Wang-Pruski G (2019). Comparative transcriptome analyses revealed different heat stress responses in high-and low-GS Brassica alboglabra sprouts. BMC Genomics.

[16] Brown PD, Tokuhisa JG, Reichelt M, Gershenzon J (2003). Variation of glucosinolate accumulation among different organs and developmental stages of Arabidopsis thaliana. Phytochemistry.

[17] Grubb CD, Abel S (2006). Glucosinolate metabolism and its control. Trends Plant Sci.

[18] Mitreiter S, Gigolashvili T (2020). Regulation of glucosinolate biosynthesis. J Exp Bot.

[19] Sønderby IE, Geu-Flores F, Halkier BA (2010). Biosynthesis of glucosinolates–gene discovery and beyond. Trends Plant Sci.

[20] Textor S, De Kraker J-W, Hause B, Gershenzon J, Tokuhisa JG (2007). MAM3 catalyzes the formation of all aliphatic glucosinolate chain lengths in Arabidopsis. Plant Physiol.

[21] Burow M, Atwell S, Francisco M, Kerwin RE, Halkier BA, Kliebenstein DJ (2015). The glucosinolate biosynthetic gene AOP2 mediates feed-back regulation of jasmonic acid signaling in Arabidopsis. Mol Plant.

[22] Huseby S, Koprivova A, Lee B-R, Saha S, Mithen R, Wold A-B, Bengtsson GB, Kopriva S (2013). Diurnal and light regulation of Sulphur assimilation and glucosinolate biosynthesis in Arabidopsis. J Exp Bot.

[23] Wang L, Liu D, Ahmed T, Chung F-L, Conaway C, Chiao J-W (2004). Targeting cell cycle machinery as a molecular mechanism of sulforaphane in prostate cancer prevention. Int J Oncol.

[24] Wittstock U, Halkier BA (2002). Glucosinolate research in the Arabidopsis era. Trends Plant Sci.

[25] Chadchawan S, Bishop J, Thangstad OP, Bones AM, Mitchell-Olds T, Bradley D (1993). Arabidopsis cDNA sequence encoding myrosinase. Plant Physiol.

[26] Xue J, Lenman M, Falk A, Rask L (1992). The glucosinolate-degrading enzyme myrosinase in Brassicaceae is encoded by a gene family. Plant Mol Biol.

[27] Vassão DG, Wielsch N, AMdMM G, Gebauer-Jung S, Hupfer Y, Svatoš A, Gershenzon J (2018). Plant defensive β-glucosidases resist digestion and sustain activity in the gut of a lepidopteran herbivore. Front Plant Sci.

[28] Nitz I, Berkefeld H, Puzio PS, Grundler FM (2001). Pyk10, a seedling and root specific gene and promoter from Arabidopsis thaliana. Plant Sci.

[29] Nakano RT, Piślewska-Bednarek M, Yamada K, Edger PP, Miyahara M, Kondo M, Böttcher C, Mori M, Nishimura M, Schulze-Lefert P (2017). PYK10 myrosinase reveals a functional coordination between endoplasmic reticulum bodies and glucosinolates in Arabidopsis thaliana. Plant J.

[30] Nakazaki A, Yamada K, Kunieda T, Sugiyama R, Hirai MY, Tamura K, Hara-Nishimura I, Shimada T (2019). Leaf endoplasmic reticulum bodies identified in Arabidopsis rosette leaves are involved in defense against herbivory. Plant Physiol.

[31] Nour-Eldin HH, Halkier BA (2009). Piecing together the transport pathway of aliphatic glucosinolates. Phytochem Rev.

[32] Nour-Eldin HH, Andersen TG, Burow M, Madsen SR, Jørgensen ME, Olsen CE, Dreyer I, Hedrich R, Geiger D, Halkier BA (2012). NRT/PTR transporters are essential for translocation of glucosinolate defence compounds to seeds. Nature.

[33] Nour-Eldin HH, Madsen SR, Engelen S, Jørgensen ME, Olsen CE, Andersen JS, Seynnaeve D, Verhoye T, Fulawka R, Denolf P, Halkier BA (2017). Reduction of antinutritional glucosinolates in Brassica oilseeds by mutation of genes encoding transporters. Nat Biotechnol.

[34] Jørgensen ME, Olsen CE, Geiger D, Mirza O, Halkier BA, Nour-Eldin HH (2015). A functional EXXEK motif is essential for proton coupling and active glucosinolate transport by NPF2. 11. Plant Cell Physiol.

[35] Li H, Yu M, Du X-Q, Wang Z-F, Wu W-H, Quintero FJ, Jin X-H, Li H-D, Wang Y (2017). NRT1. 5/NPF7. 3 functions as a proton-coupled H+/K+ antiporter for K+ loading into the xylem in Arabidopsis. Plant Cell.

[36] Yang Y, Hu Y, Yue Y, Pu Y, Yin X, Duan Y, Huang A, Yang Y, Yang Y (2020). Expression profiles of glucosinolate biosynthetic genes in turnip (Brassica rapa var. rapa) at different developmental stages and effect of transformed flavin-containing monooxygenase genes on hairy root glucosinolate content. J Sci Food Agric.

[37] Strickler SR, Bombarely A, Mueller LA (2012). Designing a transcriptome next-generation sequencing project for a nonmodel plant species1. Am J Bot.

[38] Meldau S, Erb M, Baldwin IT (2012). Defence on demand: mechanisms behind optimal defence patterns. Ann Bot.

[39] Kanehisa M, Araki M, Goto S, Hattori M, Hirakawa M, Itoh M, Katayama T, Kawashima S, Okuda S, Tokimatsu T (2007). KEGG for linking genomes to life and the environment. Nucleic Acids Res.

[40] Kanehisa M, Furumichi M, Tanabe M, Sato Y, Morishima K (2016). KEGG: new perspectives on genomes, pathways, diseases and drugs. Nucleic Acids Res.

[41] Gigolashvili T, Yatusevich R, Berger B, Müller C, Flügge UI (2007). The R2R3-MYB transcription factor HAG1/MYB28 is a regulator of methionine-derived glucosinolate biosynthesis in Arabidopsis thaliana. Plant J.

[42] Petersen B, Chen S, Hansen C, Olsen C, Halkier B (2002). Composition and content of glucosinolates in developing Arabidopsis thaliana. Planta.

[43] Burow M, Halkier BA (2017). How does a plant orchestrate defense in time and space? Using glucosinolates in Arabidopsis as case study. Curr Opin Plant Biol.

[44] Magrath R, Mithen R (1993). Maternal effects on the expression of individual aliphatic glucosinolates in seeds and seedlings of Brassica napus. Plant Breed.

[45] Kliebenstein DJ, Lambrix VM, Reichelt M, Gershenzon J, Mitchell-Olds T (2001). Gene duplication in the diversification of secondary metabolism: tandem 2-oxoglutarate–dependent dioxygenases control glucosinolate biosynthesis in Arabidopsis. Plant Cell.

[46] Field B, Cardon G, Traka M, Botterman J, Vancanneyt G, Mithen R (2004). Glucosinolate and amino acid biosynthesis in Arabidopsis. Plant Physiol.

[47] Abrahams RS, Pires JC, Schranz ME (2020). Genomic origin and diversification of the Glucosinolate MAM locus. Front Plant Sci.

[48] Liu S, Liu Y, Yang X, Tong C, Edwards D, Parkin IAP, Zhao M, Ma J, Yu J, Huang S, Wang X, Wang J, Lu K, Fang Z, Bancroft I, Yang TJ, Hu Q, Wang X, Yue Z, Li H, Yang L, Wu J, Zhou Q, Wang W, King GJ, Pires JC, Lu C, Wu Z, Sampath P, Wang Z, Guo H, Pan S, Yang L, Min J, Zhang D, Jin D, Li W, Belcram H, Tu J, Guan M, Qi C, du D, Li J, Jiang L, Batley J, Sharpe AG, Park BS, Ruperao P, Cheng F, Waminal NE, Huang Y, Dong C, Wang L, Li J, Hu Z, Zhuang M, Huang Y, Huang J, Shi J, Mei D, Liu J, Lee TH, Wang J, Jin H, Li Z, Li X, Zhang J, Xiao L, Zhou Y, Liu Z, Liu X, Qin R, Tang X, Liu W, Wang Y, Zhang Y, Lee J, Kim HH, Denoeud F, Xu X, Liang X, Hua W, Wang X, Wang J, Chalhoub B, Paterson AH (2014). The Brassica oleracea genome reveals the asymmetrical evolution of polyploid genomes. Nat Commun.

[49] Lee Y-S, Ku K-M, Becker TM, Juvik JA (2017). Chemopreventive glucosinolate accumulation in various broccoli and collard tissues: microfluidic-based targeted transcriptomics for by-product valorization. PLoS One.

[50] Li G, Quiros C (2003). In planta side-chain glucosinolate modification in Arabidopsis by introduction of dioxygenase Brassica homolog BoGSL-ALK. Theor Appl Genet.

[51] Li Z, Zheng S, Liu Y, Fang Z, Yang L, Zhuang M, Zhang Y, Lv H, Wang Y, Xu D (2021). Characterization of glucosinolates in 80 broccoli genotypes and different organs using UHPLC-triple-TOF-MS method. Food Chem.

[52] Kim JK, Chu SM, Kim SJ, Lee DJ, Lee SY, Lim SH, Ha S-H, Kweon SJ, Cho HS (2010). Variation of glucosinolates in vegetable crops of Brassica rapa L. ssp. pekinensis. Food Chem.

[53] Adams KL, Cronn R, Percifield R, Wendel JF (2003). Genes duplicated by polyploidy show unequal contributions to the transcriptome and organ-specific reciprocal silencing. Proc Natl Acad Sci.

[54] Zhang Y, Malzahn AA, Sretenovic S, Qi Y (2019). The emerging and uncultivated potential of CRISPR technology in plant science. Nature Plants.

[55] Meier K, Ehbrecht MD, Wittstock U (2019). Glucosinolate content in dormant and germinating Arabidopsis thaliana seeds is affected by non-functional alleles of classical myrosinase and nitrile-specifier protein genes. Front Plant Sci.

[56] Chen Y, Chen Y, Shi C, Huang Z, Zhang Y, Li S, Li Y, Ye J, Yu C, Li Z (2018). SOAPnuke: a MapReduce acceleration-supported software for integrated quality control and preprocessing of high-throughput sequencing data. Gigascience.

[57] Bolger AM, Lohse M, Usadel B (2014). Trimmomatic: a flexible trimmer for Illumina sequence data. Bioinformatics.

[58] Langmead B, Salzberg SL (2012). Fast gapped-read alignment with bowtie 2. Nat Methods.

[59] Li B, Dewey CN (2011). RSEM: accurate transcript quantification from RNA-Seq data with or without a reference genome. BMC Bioinformatics.

[60] Altschul SF, Madden TL, Schäffer AA, Zhang J, Zhang Z, Miller W, Lipman DJ (1997). Gapped BLAST and PSI-BLAST: a new generation of protein database search programs. Nucleic Acids Res.

[61] Ashburner M, Ball CA, Blake JA, Botstein D, Butler H, Cherry JM, Davis AP, Dolinski K, Dwight SS, Eppig JT (2000). Gene ontology: tool for the unification of biology. Nat Genet.

[62] Kanehisa M, Goto S, Kawashima S, Okuno Y, Hattori M (2004). The KEGG resource for deciphering the genome. Nucleic Acids Res.

[63] Wang L, Feng Z, Wang X, Wang X, Zhang X (2010). DEGseq: an R package for identifying differentially expressed genes from RNA-seq data. Bioinformatics.

[64] Wickham H: ggplot2: elegant graphics for data analysis: Springer; 2016.

[65] Chen C, Chen H, Zhang Y, Thomas HR, Frank MH, He Y, et al. TBtools-an integrative toolkit developed for interactive analyses of big biological data. bioRxiv. 2020:289660. 10.1016/j.molp.2020.06.009 32585190

[66] Cv M, Huynen M, Jaeggi D, Schmidt S, Bork P, Snel B (2003). STRING: a database of predicted functional associations between proteins. Nucleic Acids Res.

